# Relative vaccine effectiveness of high-dose vs. standard-dose influenza vaccine against clinical outcomes according to history of atrial fibrillation: a pre-specified analysis of the DANFLU-1 randomized trial

**DOI:** 10.1093/ehjopen/oeaf102

**Published:** 2025-08-21

**Authors:** Caroline Espersen, Niklas Dyrby Johansen, Daniel Modin, Kira Hyldekær Janstrup, Matthew M Loiacono, Rebecca C Harris, Tor Biering-Sørensen

**Affiliations:** Department of Cardiology, Copenhagen University Hospital—Herlev and Gentofte, Copenhagen, Gentofte Hospitalsvej 8, 2900 Hellerup, Denmark; Center for Translational Cardiology and Pragmatic Randomized Trials, Department of Biomedical Sciences, Faculty of Health and Medical Sciences, University of Copenhagen, Gentofte Hospitalsvej 8, 2900 Hellerup, Denmark; Department of Cardiology, Copenhagen University Hospital—Herlev and Gentofte, Copenhagen, Gentofte Hospitalsvej 8, 2900 Hellerup, Denmark; Center for Translational Cardiology and Pragmatic Randomized Trials, Department of Biomedical Sciences, Faculty of Health and Medical Sciences, University of Copenhagen, Gentofte Hospitalsvej 8, 2900 Hellerup, Denmark; Department of Cardiology, Copenhagen University Hospital—Herlev and Gentofte, Copenhagen, Gentofte Hospitalsvej 8, 2900 Hellerup, Denmark; Center for Translational Cardiology and Pragmatic Randomized Trials, Department of Biomedical Sciences, Faculty of Health and Medical Sciences, University of Copenhagen, Gentofte Hospitalsvej 8, 2900 Hellerup, Denmark; Department of Cardiology, Copenhagen University Hospital—Herlev and Gentofte, Copenhagen, Gentofte Hospitalsvej 8, 2900 Hellerup, Denmark; Center for Translational Cardiology and Pragmatic Randomized Trials, Department of Biomedical Sciences, Faculty of Health and Medical Sciences, University of Copenhagen, Gentofte Hospitalsvej 8, 2900 Hellerup, Denmark; Sanofi Vaccines, Medical Evidence Generation, Sanofi, 100 Morris St, Morristown, NJ 07960, USA; Department of Epidemiology, University of Delaware, 100 Discovery Blvd, Newark, DE 19713, USA; Sanofi Vaccines, Medical Evidence Generation, 38 Beach Road, Singapore 189767; Department of Cardiology, Copenhagen University Hospital—Herlev and Gentofte, Copenhagen, Gentofte Hospitalsvej 8, 2900 Hellerup, Denmark; Center for Translational Cardiology and Pragmatic Randomized Trials, Department of Biomedical Sciences, Faculty of Health and Medical Sciences, University of Copenhagen, Gentofte Hospitalsvej 8, 2900 Hellerup, Denmark; Department of Cardiology, Copenhagen University Hospital—Rigshospitalet, Blegdamsvej 9, 2100 Copenhagen, Denmark

**Keywords:** Atrial fibrillation, Influenza, Influenza vaccine, Mortality, Hospitalization

## Abstract

**Aims:**

Atrial fibrillation (AF) may be associated with adverse influenza-related outcomes. We assessed the relative vaccine effectiveness (rVE) of high-dose (HD-IIV) vs. standard-dose (SD-IIV) inactivated influenza vaccination against cardiovascular and all-cause hospitalizations and all-cause mortality according to history of AF.

**Methods and results:**

This was a prespecified analysis of DANFLU-1, a pragmatic, open-label, feasibility trial randomizing adults aged 65-79 years 1:1 to HD-IIV or SD-IIV during the 2021-2022 influenza season in Denmark. Baseline and endpoint data were obtained from the nationwide administrative health registries. Prespecified endpoints included cardiovascular hospitalizations and all-cause mortality occurring 14 days after vaccination until 31 May 2022. Among 12 477 randomized participants, 878 (7.0%) had AF at baseline. Participants with AF were older (73.0 ± 3.8 vs. 71.7 ± 3.9 years, *P* < 0.001), more likely to be male (70.7% vs. 51.5%, *P* < 0.001) and have concomitant comorbidities. The incidence rate of hospitalization for AF was 75.5 vs. 5.1 per 1000 person-years for individuals with vs. without AF (*P* < 0.001). HD-IIV vs. SD-IIV was associated with a lower all-cause mortality rate irrespective of AF status (AF: 9 events, rVE 54.1%, 95% CI −114.7 to 92.6% vs. no AF: 53 events, rVE 48.3%, 95% CI 6.3-72.5%, pinteraction = 0.87). HD-IIV was not associated with a lower incidence of AF hospitalization regardless of AF status (overall rVE: 29.7%, 95% CI −13.9 to 57.1, pinteraction = 0.51).

**Conclusion:**

Although DANFLU-1 was not powered for clinical endpoints, HD-IIV vs. SD-IIV was associated with lower all-cause mortality irrespective of AF status. HD-IIV compared with SD-IIV was not associated with a significantly lower incidence of AF hospitalizations regardless of AF status.

## Graphical Abstract

**Graphical Abstract:**
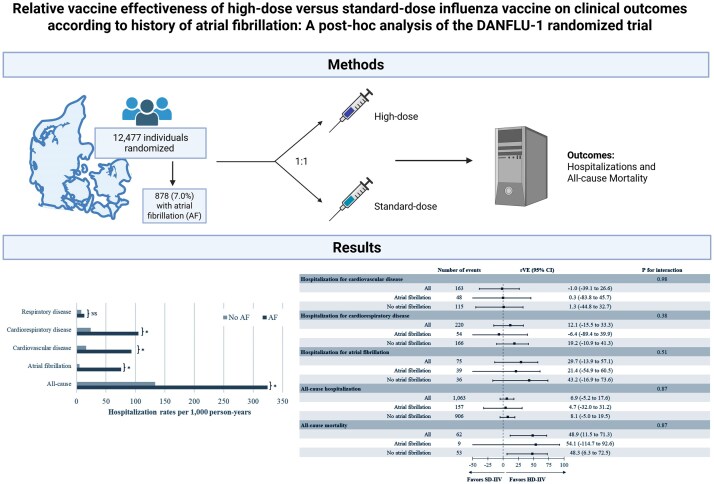

## Introduction

Influenza infection has been associated with an elevated risk of respiratory and cardiovascular events and mortality among patients with atrial fibrillation (AF).^1^ Influenza may also trigger AF development due to increased sympathetic activity and inflammatory response,^2^ which may lead to higher AF hospitalization rates. Studies have suggested a protective non-specific effect of influenza vaccination in patients with AF both with regards to stroke risk^3^ and AF prevention.^2^ However, as individuals with cardiovascular disease may exhibit sub-optimal antibody response towards influenza vaccination,^4^ this may result in reduced effectiveness of the standard-dose influenza vaccine. To improve protection against influenza and influenza-related complications for high-risk individuals, high-dose influenza vaccines were developed; in a randomized controlled trial, high-dose influenza vaccines have demonstrated superior efficacy in preventing influenza infection compared with standard-dose influenza vaccines in older adults.^5^ Moreover, individuals with AF are typically older with a higher burden of cardiovascular comorbidities^6^ and may therefore represent a population for which the high-dose influenza vaccine could have added benefit. However, little is known regarding the added benefit of high-dose compared with standard-dose influenza vaccines on important clinical outcomes among individuals with AF. DANFLU-1 (feasibility of randomizing Danish citizens ages 65–79 years to high-dose quadrivalent influenza vaccine vs. standard-dose quadrivalent influenza vaccine in a pragmatic registry-based setting) was a pragmatic, registry-based trial with the primary objective of assessing the feasibility of conducting large-scale vaccine trials within the Danish health system. The trial investigated the relative effectiveness of high-dose (HD-IIV) compared with standard-dose (SD-IIV) inactivated influenza vaccines against hospitalizations and mortality.^7^ DANFLU-1 showed that HD-IIV was associated with a lower incidence of hospitalizations for influenza or pneumonia and all-cause mortality compared with SD-IIV.^7^ In this pre-specified study, we sought to assess the relative effectiveness of HD-IIV vs. SD-IIV against clinically relevant hospitalizations and mortality according to AF status based on the DANFLU-1 trial.

## Methods

This was a *post-hoc* analysis of DANFLU-1.^7^ The study design, acquisition of registry data and primary findings of DANFLU-1 have previously been decribed.^7^ DANFLU-1 was a pragmatic, registry-based, open-label, randomized trial assessing the feasibility of randomizing Danish adults aged 65–79 years to HD-IIV vs. SD-IIV during the 2021–2022 influenza season. The study was approved by relevant authorities. The trial is registered at Clinicaltrials.gov: NCT05048589. All participants provided written informed consent prior to participation. Participant enrolment lasted from 1 October 2021 to 20 November 2021.

Participants were randomized 1:1 to high-dose quadrivalent or standard-dose quadrivalent influenza vaccine using central blocked randomization. All data were retrieved passively from nationwide registries containing routinely obtained health data, minimizing the risk of ascertainment bias. Pre-specified definitions of baseline diagnoses, medication use and outcomes based on the International Classification of Disease, 10th edition (ICD-10) and Anatomical Therapeutic Chemical Classification codes have previously been described.^7^

The following pre-specified outcomes were assessed: hospitalizations for (i) respiratory disease, (ii) cardiorespiratory disease, (iii) cardiovascular disease, (iv) AF, and (v) all causes, and (vi) all-cause mortality. The follow-up period for clinical outcomes spanned from 14 days post-vaccination until 31 May 2022.

### Statistical analysis

All analyses were performed according to the intention-to-treat principle. We calculated relative vaccine effectiveness (rVE) comparing HD-IIV vs. SD-IIV using first events according to the following formula:


rVE=(1−RR)*100%

95% Confidence intervals were calculated using the Clopper–Pearson method. We assessed for effect modification by AF status using log-binomial regression analysis. Hospitalization rates (first events only) per 1000 person-years were compared in patients with and without AF. A *P*-value <0.05 was considered statistically significant. SAS Software, version 9.4 (SAS Institute) and Stata MP, version 17.0 (StataCorp) were used for the statistical analysis.

## Results

Among 12 477 included participants, 878 (7.0%) had AF at baseline. Overall, participants with AF were older (73.0 (standard deviation (SD) 3.8) vs. 71.7 (SD 3.9) years, *P* < 0.001) and more likely to be male (621 (70.7%) vs. 5976 (51.5%), *P* < 0.001) and have concomitant comorbidities, including ischaemic heart disease (152 (17.3%) vs. 810 (7.0%), *P* < 0.001), hypertension (750 (85.4%) vs. 5719 (49.3%), *P* < 0.001), diabetes (131 (14.9%) vs. 1031 (8.9%), *P* < 0.001) and chronic lung disease (101 (11.5%) vs. 749 (6.5%), *P* < 0.001) compared with participants without AF. Median CHA_2_DS_2_-VASc score in patients with AF was 3 (IQR 2–4).

**Figure 1:**
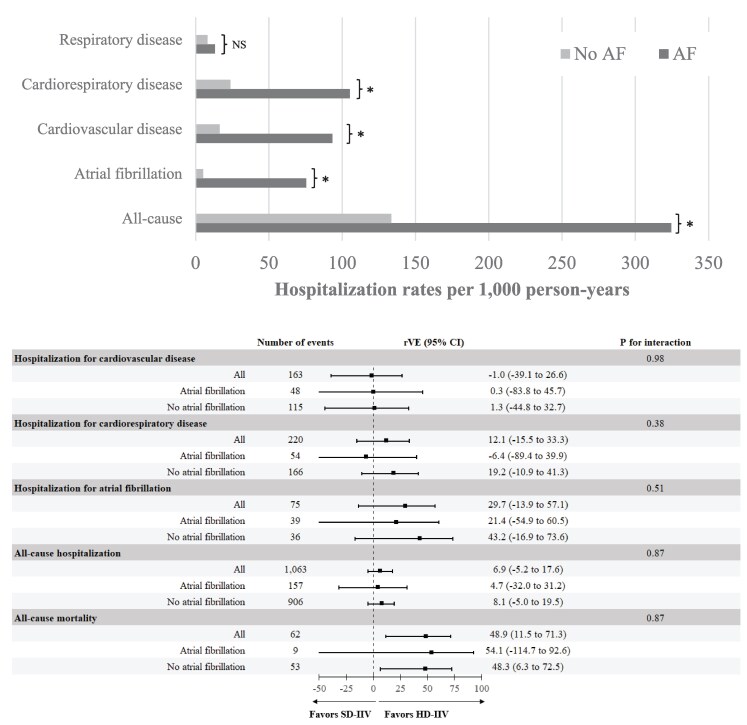
Hospitalization rates according to AF status (top panel) and the rVE of HD-IIV compared with SD-IIV overall and according to AF status (lower panel). Top panel: a bar graph depicting the overall hospitalization rates according to AF status. Lower panel: Forest plot depicting rVE and 95% confidence intervals calculated using the Clopper–Pearson method for the individual outcomes according to AF status. Tests for interaction were performed using binomial regression models. *P < 0.001. rVE, relative vaccine effectiveness; AF, atrial fibrillation.

The incidence rates of hospitalizations per 1000 person-years were higher for all clinical outcomes among those with AF compared with those without except for respiratory hospitalizations (*Figure 1*). Specifically, the incidence rate of hospitalization for AF was 75.5 per 1000 person–years in individuals with AF vs. 5.1 per 1000 person–years in individuals without AF (*P* < 0.001). The corresponding incidence rates for hospitalization for cardiovascular disease were 93.3 vs. 16.4 per 1000 person–years for individuals with and without AF, respectively (*P* < 0.001).

HD-IIV was associated with a lower all-cause mortality rate compared with SD-IIV, regardless of AF status (rVE: 48.9%, 95% CI 11.5% to 71.3% for total population, rVE 54.1%, 95% CI −114.7 to 92.6% in individuals with AF and rVE 48.3%, 95% CI 6.3% to 72.5% in individuals without AF, *P*-value for interaction = 0.87) (*Figure 1*). AF at baseline did not modify rVE of HD-IIV vs. SD-IIV for any of the clinical outcomes assessed. In addition, though there was a trend toward a lower incidence of AF hospitalizations among HD-IIV randomized participants, there were no significant differences in the hospitalization endpoints across randomization groups, overall and regardless of AF status (*Figure 1*).

## Discussion

Although DANFLU-1 was not powered for clinical outcomes, the overall results suggested that HD-IIV vs. SD-IIV was associated with a lower incidence of hospitalizations for influenza or pneumonia and all-cause mortality. This pre-specified analysis expands on these findings by showing no effect modification by AF status at baseline for all-cause mortality, suggesting consistent vaccine effectiveness of HD-IIV vs. SD-IIV regardless of AF status.

Overall, despite a higher prevalence of comorbidities, participants with AF did not accrue a high number of respiratory events during the relatively short follow-up period. Instead, patients with AF were more likely to be hospitalized for cardiovascular disease, including AF hospitalizations. Although HD-IIV trended in favour of a lower incidence of AF hospitalizations, we did not find a significant association between HD-IIV vs. SD-IIV and AF hospitalizations in this study.

Prior studies have suggested that AF may be associated with worse outcomes with influenza infection including increased mortality risk,^1^ likely explained by multi-factorial mechanisms including increased sympathetic and inflammatory response associated with both AF and influenza. In this exploratory analysis, HD-IIV offered improved protection against all-cause mortality irrespective of AF status, suggesting a similar effectiveness and benefit of HD-IIV vs. SD-IIV among individuals with AF as for individuals without AF. As AF is the most common sustained cardiac arrhythmia affecting nearly one in three to five individuals worldwide,^8^ providing HD-IIV vaccine may be a relatively inexpensive prevention strategy to further decrease mortality risk in this population. However, further adequately powered trials are warranted to test this hypothesis. We plan to conduct a pre-specified secondary analysis of the clinically powered DANFLU-2 trial (A Pragmatic Randomized Trial to Evaluate the Effectiveness of High-Dose Quadrivalent Influenza Vaccine vs. Standard-Dose Quadrivalent Influenza Vaccine in Older Adults (Clinicaltrials.gov: NCT05517174)) assessing the relative effectiveness of HD-IIV compared with SD-IIV in older adults over three influenza seasons, focusing on individuals with AF.

### Limitations

This was a pre-specified analysis of DANFLU-1, which was not powered for clinical outcomes but rather for feasibility, and as such, all outcome analyses are considered exploratory and hypothesis-generating only. Moreover, the trial was conducted in Denmark during the northern hemisphere influenza season and the findings may therefore not be generalizable to other populations with differing demography and circulating virus strains.

## Conclusion

Although DANFLU-1 was not powered for clinical endpoints, high-dose influenza vaccine was associated with lower all-cause mortality compared with standard-dose influenza vaccine regardless of AF status at baseline in this pre-specified analysis. We did not find a significant association between high-dose vs. standard-dose influenza vaccine and AF hospitalizations in this study.

## Data Availability

Data from the nationwide Danish administrative health registries are subject to Danish legislation and can only be made available to a third party under certain conditions. Please contact the corresponding author in case of any inquiries.

## Lead author biography

Dr. Caroline Espersen is a PhD fellow at the Department of Cardiology, Copenhagen University Hospital—Herlev and Gentofte, Copenhagen, Denmark. She has worked with cardiovascular research for several years since medical school, with prior research stays at Brigham and Women’s Hospital, Harvard Medical School as a Fulbright Scholar, and more recently at the University of California, San Francisco. She has worked with observational studies, registry-based studies, and clinical trials in cardiovascular disease, with a primary focus on acute heart failure and atrial fibrillation. She currently co-ordinates a large randomized controlled trial in atrial fibrillation and serves as a Science Ambassador for the Danish Cardiovascular Academy. She was recently awarded the Danish Cardiovascular Academy Rising Star Award for her contributions to the cardiovascular research field.
